# Dens Evaginatus: A Problem-Based Approach

**DOI:** 10.1155/2015/393209

**Published:** 2015-12-08

**Authors:** A. Ayer, M. Vikram, P. Suwal

**Affiliations:** ^1^Department of Conservative Dentistry & Endodontics, B.P. Koirala Institute of Health Sciences, Dharan 56700, Nepal; ^2^Department of Prosthodontics, B.P. Koirala Institute of Health Sciences, Dharan 56700, Nepal

## Abstract

Dens evaginatus is an uncommon developmental anomaly of human dentition characterized by the presence of tubercle on the occlusal surface of mandibular premolars and lingual surface of anterior teeth. Due to occlusal trauma this tubercle tends to fracture thus exposing the pathway to the pulp chamber of teeth. This case report is about the presentation of dens evaginatus in mandibular premolars bilaterally; among them tooth 44 was associated with chronic apical periodontitis. Fractured tubercle of three premolars was sealed with composite resin. Root canal treatment was performed with tooth 44. Routine endodontic treatment did not result in remission of infection. Therefore, culture and sensitivity tests were performed to identify the cause and modify treatment plan accordingly. Triple antibiotic paste was used as an intracanal medicament to disinfect the root canal that resulted in remission of infection.

## 1. Introduction

Dens evaginatus (DE) or evaginated odontoma is a developmental anomaly characterized by the presence of an accessory cusp, abnormal tubercle, or elevation that occurs in human dentition. It consists of enamel covering a dentinal core that usually contains pulp tissue. The presence of pulp within the cusp-like tubercle has clinical significance and distinguishes it from supplemental cusps, such as cusp of carabelli [1]. Early detection and management of this condition are important because trauma during mastication causes fracture or wear of the tubercle that leads to necrosis of pulp and periapical infection. This condition is predominantly found on the occlusal surface of mandibular premolars [2] and lingual surface of anterior teeth (mainly maxillary lateral incisors) [3]. Prevalence of DE studied in several population ranges from 1% to 4% [4]; the prevalence was found in approximately 2% of Asian descent population [5]. Higher rates of occurrence were reported among the Chinese population, 1.29%–3.6% [6]. It is usually observed as bilateral, symmetric distribution, with a slight sexual predilection for females.

## 2. Case Presentation

**Figure 1:**
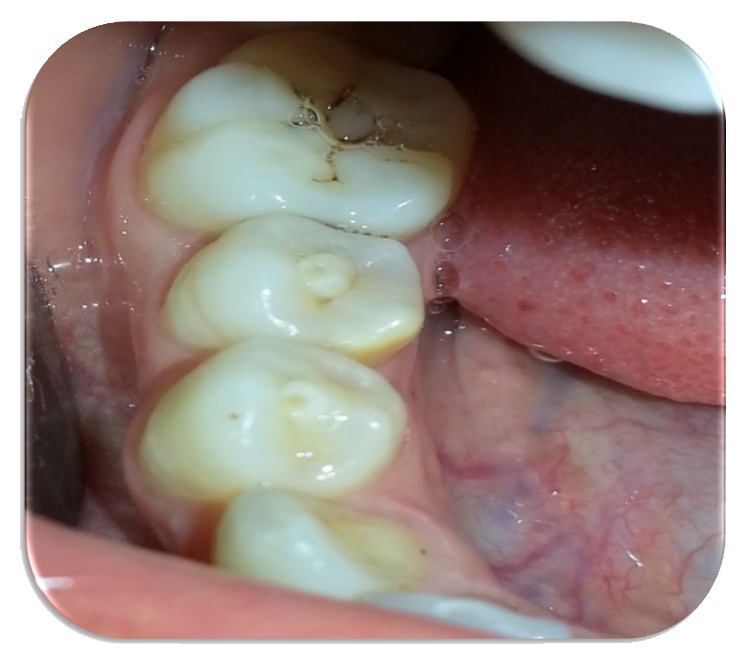
Occlusal tubercle in the mandibular premolars.

**Figure 2:**
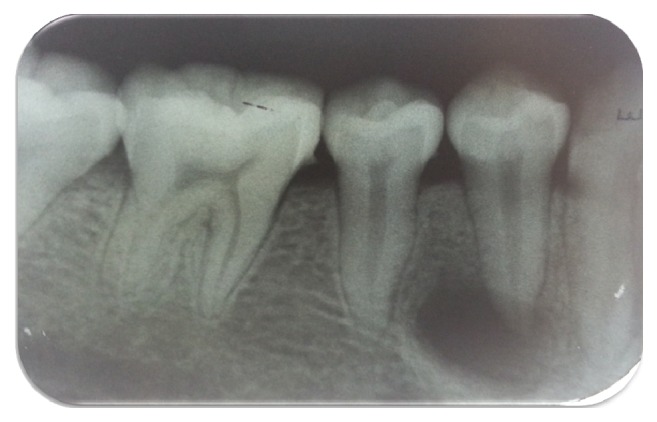
Diagnostic IOPAR, periapical radiolucency with respect to 44.

**Figure 3:**
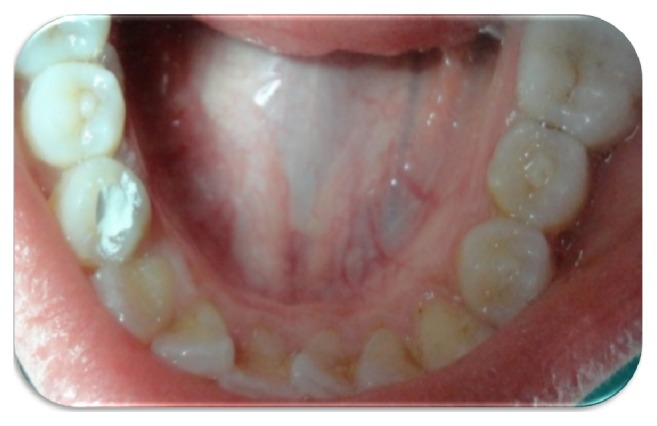
After access opening. Dens evaginatus in right and left premolars.

A 20-year-old female attended at the department. The patient was concerned about the “bubble on her gums.” On examination patient had fair oral hygiene and occlusal tubercle present in mandibular premolars (Figure 1). Pus discharge from sinus tract adjacent to tooth 44 was observed. Intraoral Periapical Radiograph (IOPAR) revealed periapical radiolucency with respect to 44 (Figure 2). Electrical pulp sensitivity test shows vital mandibular premolars except 44. No systematic and congenital disease was seen. Root canal therapy (RCT) of tooth 44 and adjustment of occlusal tubercle of teeth 34, 35, and 45 were planned (Figure 3).

**Figure 4:**
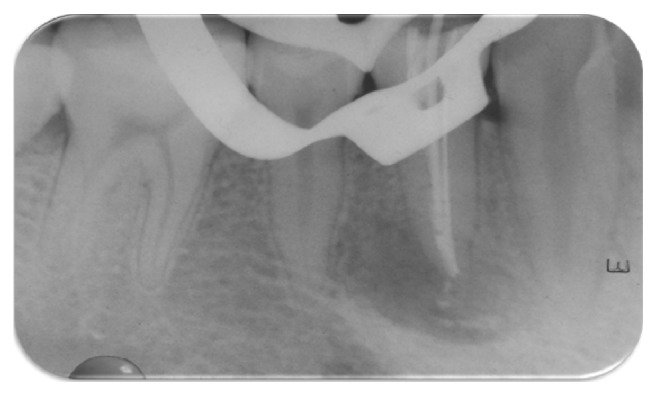
Placement of calcium hydroxide (Metapex) intracanal medication.

**Figure 5:**
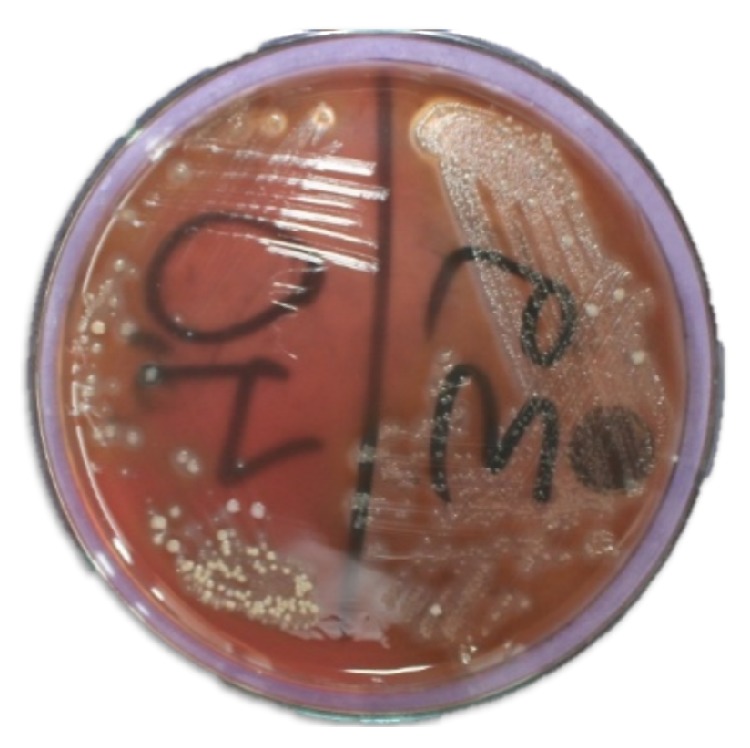
Microbiological culture showing gram positive cocci in clusters and in pair and short chains.

**Figure 6:**
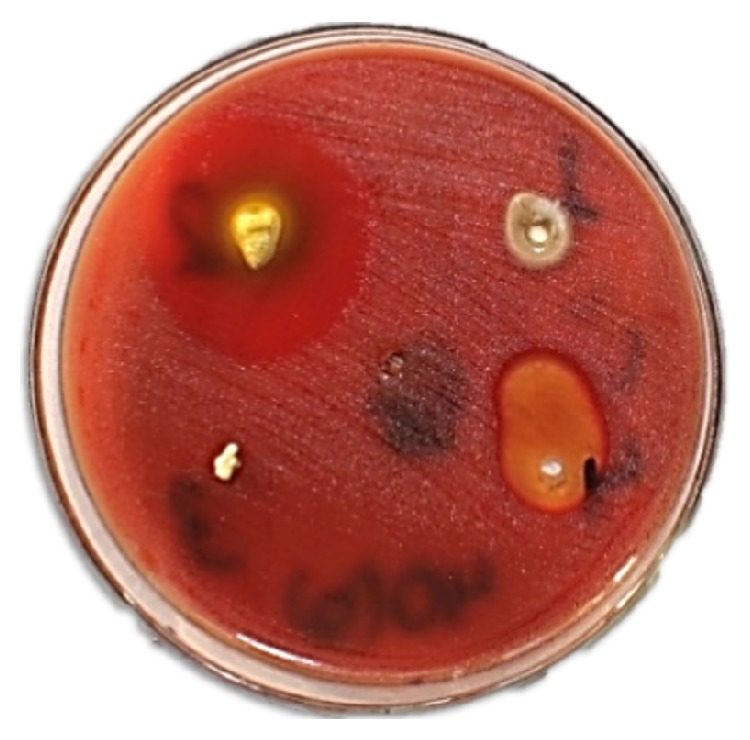
Culture and sensitivity test: triple antibiotic paste with largest inhibition zone in upper left region.

**Figure 7:**
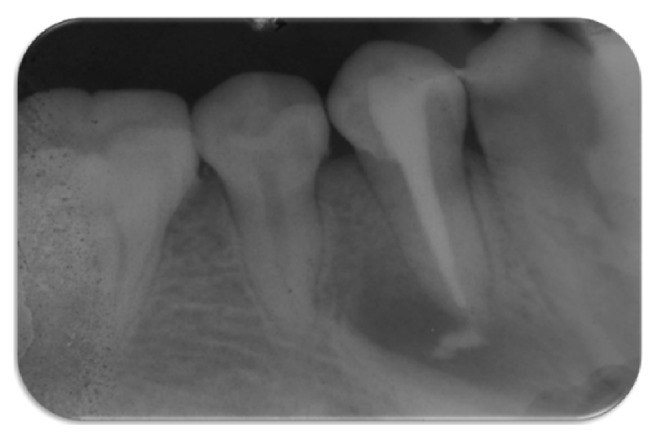
Obturation with gutta-percha and endodontic sealer.

**Figure 8:**
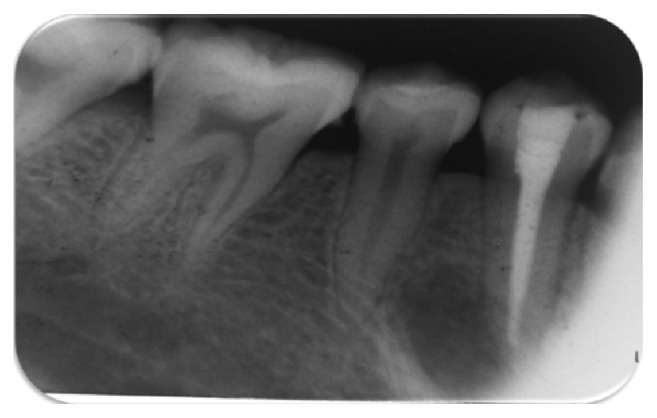
Six-month follow-up.

A slight reduction of opposing tooth contact and composite restoration of teeth 34, 35, and 45 after preparation of shallow cavities were done. Access cavity preparation, shaping, and cleaning were performed on tooth 44. Canal shaping was done according to “crown-down” instrumentation technique. The apical preparation of the canal was enlarged till no. 40 k file (Mani, Inc., Japan). During instrumentation, the canals were irrigated copiously with 2.5% sodium hypochlorite solution and root canal conditioner (Glyde File Prep, Dentsply, Maillefer, USA). Drying of the canal was done with absorbent paper points (Dentsply, Maillefer). Calcium hydroxide root canal dressing was performed and changed every 10 days followed by frequent root canal irrigation with chlorhexidine for the period of two months (Figure 4); the access cavity was temporized with the intermediate restorative material (IRM, Dentsply Caulk, Milford, USA). Despite the meticulous debridement of the root canal, the signs of healing were not appreciated. There was the persistence of sinus tract and presence of weeping canal. Culture and sensitivity tests were performed. In the culture media growth of* Enterococcus faecalis* and* Streptococcus* species was observed (Figure 5). A sensitivity test was performed using growth media and different intracanal medicaments: triple antibiotic paste (ciprofloxacin, minocycline, and metronidazole mixed in propylene glycol in a ratio of 7 : 4) [7], calcium hydroxide, chlorhexidine, and calcium hydroxide with iodoform (Metapex, META Biomed Co. Ltd., Korea). The triple antibiotic paste was seen to have the largest inhibition zone (Figure 6). Thus, we used triple antibiotic paste as an intracanal medicament and replaced the medicament every 15 days for a period of 3 months. After 3 months, the tooth was asymptomatic and sinus tract disappeared. The tooth was obturated with gutta-percha points (Dentsply, Maillefer) using AH Plus (Dentsply, Konstanz, Germany) root canal sealer followed by restoration with composite resin (Figure 7). Follow-up evaluation after six months revealed progressive healing of the lesion (Figure 8).

## 3. Discussion

Dens evaginatus is an anomaly of considerable clinical significance, often causing occlusal interference. Maintaining clean area between the nodule and the tooth is difficult, and caries is often found. There are high possibilities of pulp exposure during early phases of root development, resulting in pulp necrosis and incomplete root formation. The unusual tubercle or elevation on the tooth surface is the usual presentation but sometimes, due to fracture or attrition, no external evidence of the malformation may be evident. Thus, early detection of these conditions is important so that preventive management can be started as early as possible. In the tooth/teeth with DE with vital pulp, selective reduction of the opposing occluding teeth can be done or, in a situation where the tubercle has fractured, it can be sealed with resin. In the case of DE with pulp exposure during the early phase of root development, mineral trioxide aggregate (MTA) pulpotomy is suggested. If the pulp is necrotic, MTA root end barrier in the case of the immature apex and conventional root canal treatment should be performed on the mature tooth [1].

In the presented case, when the long-standing chronic apical periodontitis did not respond to the chemomechanical preparation and calcium hydroxide interappointment therapy, we performed microbiological culture in which growth of* Enterococcus faecalis* and* Streptococcus* species was observed. The microbial species detected in the culture has the ability of resisting treatment and causing secondary infection which then becomes persistent [8]. Another important aspect is the continued presence of symptoms and interappointment flare-up. Survival and growth of microorganisms in the culture indicate their resistance to treatment measures and the ability to adapt to the harsh environmental conditions in instrumented root canal. This represents a source of continual aggression in the periapical region. Secondary intraradicular infection is one of the major causes of endodontic treatment failure [8].* E. faecalis* has been commonly recovered from cases treated in multiple visits and/or in teeth left open for drainage [8].

Sensitivity tests were performed to select appropriate intracanal medicament. Interappointment antimicrobial medication can be advantageous to curtail bacterial regrowth and possibly even improve bacterial suppression [9]. Studies reported the clinical effectiveness of triple antibiotic paste in cases of apical periodontitis [10, 11]. The antibiotic combination was reported to be more effective against mixed bacterial flora as in infected root canal tested when compared with calcium hydroxide, iodine potassium iodide, or iodoform [12]. Gomes-Filho et al. [13] evaluated the response and concluded that triple antibiotic paste is a biocompatible intracanal medicament. A study conducted by Windley et al. [10] observed that the use of triple antibiotic paste following irrigation resulted in significant reduction in bacteria compared to that of irrigation with sodium hypochlorite alone. Studies have demonstrated that predictable disinfection of the root canal system can be achieved by proper antimicrobial intracanal medication [14].

## 4. Conclusion

The presence of communication due to the tubercle opening may contribute to harboring more virulent microorganisms that succeeds in colonizing the canal and thus resisting treatment [8]. Chronic apical periodontitis due to dens evaginatus may require special therapeutic strategies to eradicate the infection. A long-term follow-up visit is advised for inspection. The clinician should be aware of such anomalies and their consequences so that proper treatment modality can be instituted. Appropriate laboratory investigations also become of paramount importance in such cases and the judicious use of intracanal medicament will lead to a better outcome. Early detection and careful treatment planning are needed to prevent further complication of the condition.
